# Intermediate host recognition abilities of *Fasciola hepatica* miracidia

**DOI:** 10.1186/s13071-025-07125-y

**Published:** 2025-11-25

**Authors:** Natasha Sharma, Tanapan Sukee, Scott F. Cummins, Bonnie L. Webster, Winston F. Ponder, Anson V. Koehler, Neil D. Young

**Affiliations:** 1 Melbourne Veterinary School, The University of Melbourne, Parkville, VIC Australia; 2 Centre for Bioinnovation, University of Sunshine Coast, Sippy Downs, QLD Australia; 3 Natural History Museum, London, UK; 4 Australian Museum, Sydney, NSW Australia

**Keywords:** Parasite selectivity, Attachment, Fasciola, Native, Invasive, Lymnaeid, Host-seeking behaviour

## Abstract

**Background:**

*Fasciola hepatica*, the causative agent of fascioliasis in sheep and cattle, requires a compatible snail intermediate host to complete its life cycle. The aquatic larval stage of this parasite is well-adapted for host-finding, with chemotactic abilities that enable it to sense potential host biomolecules. The extent of intermediate host recognition, particularly at the species level, and the downstream correlation with successful attachment has not been explored. This study investigated the ability of *F. hepatica* miracidia to distinguish between native and invasive host and non-host freshwater snail species during the host-finding and host-attachment phases.

**Methods:**

Quantitative and qualitative measurements of miracidial behaviour were compared pre- and post-exposure with snail-conditioned water (SCW) from both native and invasive host snails (lymnaeids *Austropeplea* cf. *brazieri* and *Pseudosuccinea columella*) and non-host snails (the lymnaeid *Bullastra lessoni* and the physid *Physa acuta*). Miracidia were also exposed to live snails of each representative species to ascertain whether host-finding correlates with successful miracidial host-attachment.

**Results:**

Miracidia displayed clear shifts in movement profiles post-exposure to SCW, with no qualitative or quantitative differences observed in the behavioural response to different snail species. When exposed to live snails, miracidia were more likely to attach to both host and non-host native species (*A.* cf. *brazieri* and *B. lessoni*) compared with invasive snail species (*P. columella* and *P. acuta*). Among invasive snails, miracidia had a higher rate of successful attachment with *P. columella* (host) than with *P. acuta* (non-host).

**Conclusions:**

The miracidia of *F. hepatica* exhibit analogous host-finding responses post-exposure to SCW, regardless of which snail species they are exposed to. Host-finding responses do not correlate with miracidial ability to attach to the snail tissue or with the established host status of the respective snail species. These results provide an insight into host-finding preferences of *F. hepatica* within the Australian context and lay an important foundation for further exploration into intermediate host–parasite interactions and their mechanisms of action.

**Graphical Abstract:**

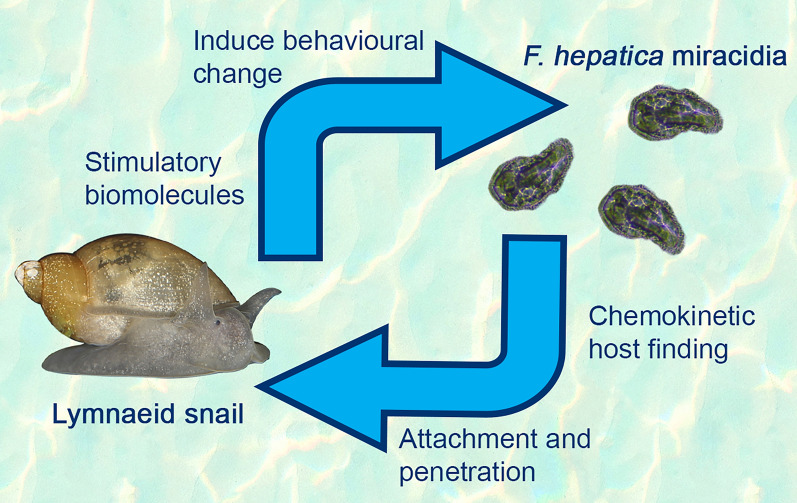

**Supplementary Information:**

The online version contains supplementary material available at 10.1186/s13071-025-07125-y.

## Background

Fascioliasis (liver fluke disease) is a foodborne zoonotic disease that impacts the production of more than 300 million animals and the health of an estimated 17 million people globally [1, 2]. Pathogenic fascioliasis is caused by digenean trematode parasites in the family Fasciolidae, namely *Fasciola hepatica* and *Fasciola gigantica* [3]. In Australia, *F. hepatica* is the exclusive causative agent of fascioliasis, originally introduced to the country in the 1700s–1800s during colonisation [4]. This disease accounts for millions of dollars in annual losses to the Australian livestock industry, with an estimated 46 million animals grazed in pastures where liver flukes are now endemic [5]. Current disease mitigation strategies rely heavily on the use of anthelmintic drugs, with triclabendazole being the gold-standard treatment for both immature and adult stages [6]. However, a sole reliance on drug administration to manage fascioliasis has led to the emergence of resistant parasite populations, first identified over three decades ago in Australian livestock [7]. Despite this, there are no viable large-scale alternative control methods, and consequently, mass drug administration remains common practice [8, 9]. Novel strategies for disease control may be discovered through a deeper understanding of the parasite’s biology, particularly the critical stages of its life cycle within intermediate host snails.

The life cycle of *F. hepatica* involves several complex developmental stages, beginning with the adult flukes releasing eggs via the faeces of the mammalian definitive host. Eggs embryonate in the environment and complete maturation within 12–16 days. Upon exposure to light and fresh water, egg hatching is stimulated [10, 11]. Small (~130 μm) free-swimming miracidia are released into an aquatic environment, where they need to find and infect a suitable host snail [12]. After successful attachment and penetration of snail soft tissue, *F. hepatica* develops into sporocysts and rediae, which produce cercariae through asexual reproduction. Hundreds of infective cercariae are then released into the water, where they encyst on surrounding vegetation and are consumed by their definitive host [13]. While the adult stage of *F. hepatica* can infect most mammals [14], this species of liver flukes can only establish infections within several specific species of lymnaeid freshwater snails [15]. Understanding compatibility within the lymnaeid snail–*Fasciola* system is of great epidemiological importance for disease monitoring and control [13, 16–20]. However, the requirements for successful parasite proliferation in this system are not well-defined, and the precise properties of *F. hepatica* miracidia that govern host selection remain unknown.

*Fasciola hepatica* miracidia have limited glycogen stores and a short lifespan of only 8–24 h, with viability diminishing as they age [21, 22]. To maximise potential host-finding efficiency and minimise energy expenditure during this critical life cycle stage, miracidia utilise geotropic, phototropic and chemotactic mechanisms to locate snails, which typically reside at pond and ditch edges [23]. Due to their short infective window, it would be advantageous for the miracidia to distinguish between intermediate host and non-host snail species to avoid unnecessarily expending their limited resources and ensure their transmission success. Parasites are unable to complete their development and be transmitted through non-host species, although invasion may still occur. While the attraction of trematode miracidia to snails has been conclusively demonstrated, no correlation between attraction and host compatibility has been definitively established [24]. Host-finding ability has primarily been studied in the miracidia of trematode parasites in the family Schistosomatidae, the causative agents of schistosomiasis [16]. *Schistosoma mansoni* miracidia have been observed to chemotactically locate and infect host snails at distances of up to 9 m in still water and 97 m in running water [25]. Evidence of host species recognition has been identified in the miracidial stage of some *S. mansoni* strains. For example, an Egyptian strain of *S. mansoni* showed strong selectivity for the host planorbid snail *Bulinus alexandrina*; however, a Brazilian strain responded equally to both host and non-host snails [26, 27]. To date, studies have demonstrated that *F. hepatica* miracidia prefer water conditioned by host snails over water from other aquatic organisms, including co-occurring snails [28, 29]. While informative, these observations were not definitive in explaining the extent of the difference in host preference and only considered the European context. Despite the importance of fascioliasis in Australia, no comprehensive exploration of miracidia host preferences has been undertaken.

This study aimed to determine whether *F. hepatica* miracidia display differences in host-finding behaviour across four snail species – native host, native non-host, invasive host and invasive non-host – to further elucidate the parasite’s host recognition abilities. The chosen snail species are prolific in fascioliasis-affected regions of Australia, and their host status with regard to permitting the successful development of *F. hepatica* has been well-studied and clearly identified [5]. *Austropeplea* cf. *brazieri* is a native host lymnaeid species, *Pseudosuccinea columella* is an invasive host lymnaeid, *Bullastra lessoni* is a native non-host lymnaeid and *Physa acuta* is an invasive, non-host physid species [5, 30, 31]. *Fasciola hepatica* miracidia behaviour was tested in response to conditioned water from all four snail species. Additionally, live snails from all groups were exposed to free-swimming *F. hepatica* miracidia to identify any differences in attachment rates between native and invasive host and non-host species, and whether this was correlated with behavioural responses.

## Methods

### Animal husbandry and preparation of snail-conditioned water (SCW)

Colonies of all four snail species, *Austropeplea* cf. *brazieri*, *P. columella*, *B. lessoni* and *P. acuta*, were maintained in aquaria at the University of Melbourne. The *A.* cf. *brazieri* colony has been maintained for several years as a laboratory strain, originally obtained from Werribee South, Victoria [32]. *Physa acuta* (collected from Werribee South, Victoria), *P. columella* (Darwin, Northern Territory) and *B. lessoni* (Wyong, New South Wales) were all first- or second-generation populations originating from field-caught snails kept for several months in laboratory conditions. All snails were raised in a standardised solution of artificial pond water [iron chloride (0.25 g/L), calcium chloride (12.90 g/L), magnesium sulphate (12.90 g/L) and phosphate buffer (261 mmol/L)]. Aquaria were maintained at 21 °C, and snails were fed ad libitum on algae wafers (Hikari, Japan) and dehydrated baby spinach leaves.

Snail-conditioned water (SCW) was prepared by immersing three to four uninfected adult snails (~10–15 mm in size) of each species in separate 60 mm plastic Petri dishes (Merck, Germany) containing ~10 mL artificial pond water. Due to different snail sizes between species, the number of snails was standardised on the basis of the area of the dish they occupied. When extended from the shell, the snail-foot surface area covered approximately one quarter of a dish. Snails were incubated at room temperature for 2 h. After incubation, 1 mL of SCW was aspirated within 1 cm of the snail’s foot, transferred to microfuge tubes and vortexed briefly before use. All SCW was used within 1 h of preparation.

### Collection of *Fasciola hepatica* miracidia

Liver fluke eggs were obtained via sedimentation [33] from the gall bladders of ten condemned sheep livers from an abattoir in Victoria, Australia. The gall bladders were severed, and the bile from all the organs was drained and pooled into a clean 3 L beaker. The eggs were washed with tap water and left to settle at the bottom of the container, and the water containing extraneous materials was removed. This process was repeated several times until the water was clear after washing. The eggs were stored in the dark at 4 °C. A 30 mL aliquot of eggs was then transferred to a 550 mL plastic culture flask (TPP, Switzerland) with artificial pond water and incubated in the dark at 25 °C for 2 weeks until fully embryonated. Hatching was induced by transferring embryonated eggs into a 250 mL conical filtration flask (Merck, Germany) containing artificial pond water. A bright white light was applied to the flask opening for 3–5 min, stimulating miracidia to hatch and swim to the water’s surface. Miracidia were collected using plastic tubing attached to the flask’s filtration arm. The tubing was positioned under a light source to phototactically attract and concentrate miracidia into a smaller water volume. Collected miracidia were transferred to microfuge tubes, and their density was adjusted via dilution to approximately 20–30 miracidia per 100 µL. Miracidial counts were performed manually using light microscopy.

### Behavioural assay

Aliquots of 150 µL of newly hatched miracidia in artificial pond water from microfuge tubes were added to wells of a 24-well plate (Corning, USA). Each treatment and control group included five biological replicates. Miracidia were recorded using an eyepiece camera at 25× magnification (Dino-eye, ANMO Electronic Corporation, Taiwan) attached to a stereo dissecting microscope (Olympus, Japan) [34] for 1 min in artificial pond water alone, followed by 1 min after the addition of 50 µL of SCW from each snail species. A total of 50 µL of fresh artificial pond water was added to the negative control groups. The movement of each miracidium that crossed the field of view of the camera was recorded. If a miracidium left the field of view and returned, it was treated as a new track; therefore, the data obtained are a representation of the population of miracidia, and not each miracidium.

Videos were processed and analysed using the linear assignment problem (LAP) tracker of the TrackMate plugin (Version 7) [35, 36] within the FIJI imaging software (Version 2.14/1.54f) [37]. Based on the size and velocity of miracidia, the TrackMate settings selected were pixel size = 7, quality filter = 2 and gap distance = 60. Detected miracidia were recorded as spots, and the linkage of their movement across frames was reported as tracks in units per second (u/s). Features obtained through the software were track velocity (distance between two recorded spots divided by frame rate), maximum distance travelled (the straight-line distance between the first spot and the furthest spot identified in the track), confinement ratio (efficiency of track displacement), mean straight-line speed (net displacement between the first and last spot divided by the track total time), linearity of forward progression (ratio between the mean straight-line speed and the track mean speed) and mean directional change (the angle between two succeeding links, averaged over all the links of a track) [35].

Tracks that were visually confirmed in the recording to have been generated by debris were either recorded as very fast (due to slight vibrations of the water) or very slow, and they were isolated to one area of the well. Based on this, only tracks with average velocities between 2 and 20 u/s and total distances travelled ≥ 20 units were retained after TrackMate data were imported into R (Version 4.4.0, “Puppycup”) [38]. Track numbers were normalised and balanced to ensure the same number of tracks were being compared amongst all replicates. The replicate with the lowest number of tracks was identified (21 tracks), and this number of tracks was randomly selected from each replicate using the sample function in R. Principal component analysis (PCA) was performed using the packages *stats*, *factoextra* and *corrplot* [38–40]. The analysis identified features contributing the most variation within the multivariate dataset, and the highest contributors were selected for subsequent analyses. These features were identified by calculating the total variation contributed by each variable for the two highest contributing dimensions. These figures were generated by adding together the contribution of each variable, weighted by the amount of variation explained by each dimension. The normality of each key track feature was assessed using the Shapiro–Wilk test [41]. Finally, pairwise Mann–Whitney *U* tests were applied to the selected features to compare metrics of behavioural change before and after SCW exposure.

### Attachment assay

Due to the speed of miracidia, highly accurate counts were difficult to obtain through real-time observation alone. Therefore, miracidia were recorded in each well before the addition of a snail and after removal of a snail for 30 s under a stereo microscope at 25× magnification (Leica Microsystems, TL5000 Ergo Transmitted Light Base). This video footage was analysed by pausing the recording five times to manually count the number of miracidia in frame, to ensure any miracidia that were overlapping or caught in a light spot were not missed. These counts were averaged to generate the final numbers.

Approximately 17–27 newly hatched miracidia were added to each well of a new 24-well plate containing 150 µL of artificial pond water, with four biological replicates for each of the four snail species being tested. Snails were submerged in carbonated water for 4–5 min at room temperature, as the hypoxic conditions cause the animals to become temporarily immobilised [42]. Snails were then quickly rinsed in artificial pond water and added to a well containing miracidia. Each snail was kept in the well for 30 min to allow sufficient time for miracidia to attach to the soft tissue [43]. Snails were removed, and the number of miracidia remaining in the wells was counted. A reduction in the number of miracidia was attributed to successful attachment.

## Results

### Qualitative observations of miracidial behaviour change in response to snail-conditioned water

**Fig. 1:**
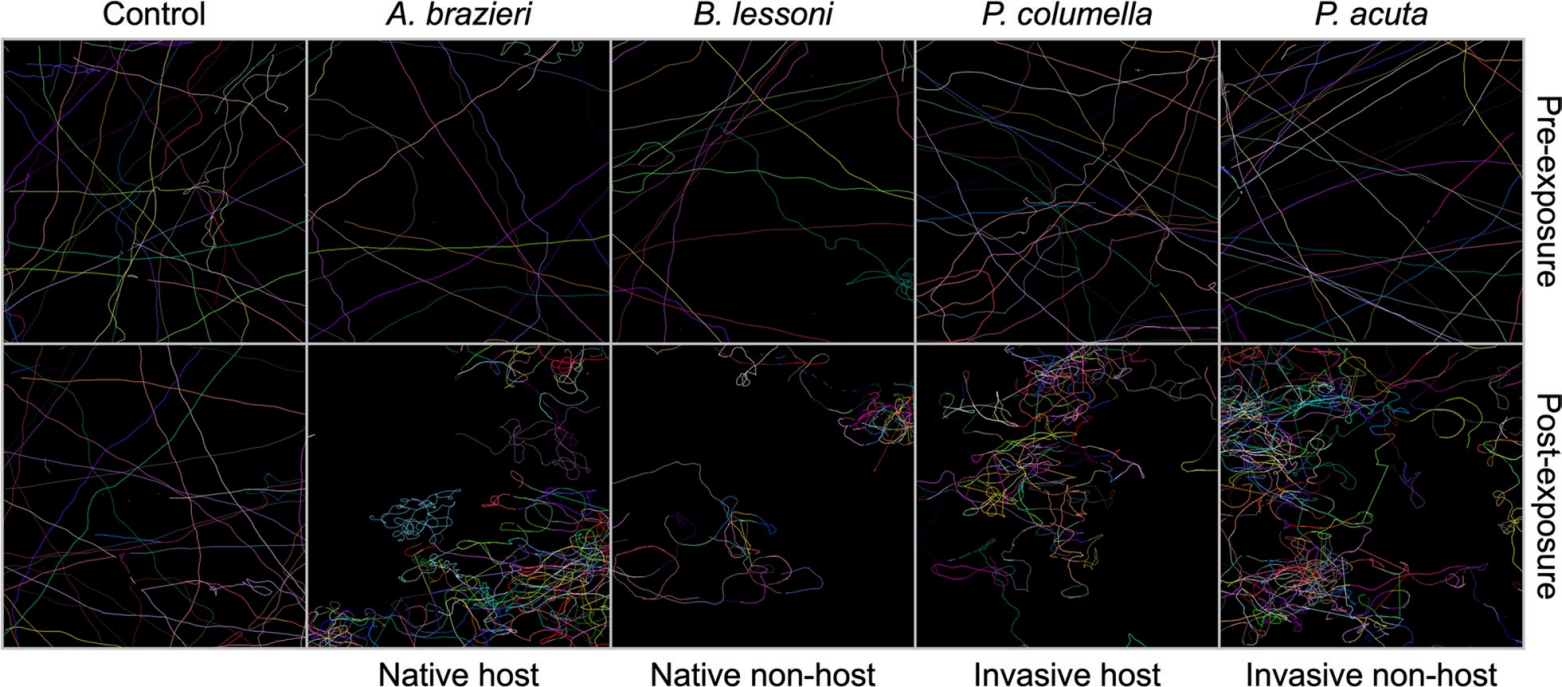
Representative tracks of *Fasciola hepatica* miracidia generated for 1 min in artificial pond water only (pre-exposure), and 1 min post-exposure to either snail-conditioned water from native host *Austropeplea* cf. *brazieri*, native non-host *Bullastra lessoni*, invasive host *Pseudosuccinea columella*, invasive non-host *Physa acuta* or artificial pond water only (negative control). Each tracking map is one well of ~35 miracidia. Tracking maps were generated using Trackmate LAP tracker

Qualitative changes in miracidia swimming behaviour were compared before and after exposure to SCW derived from the four representative lymnaeid snail species, or to artificial pond water only (Fig. 1). Representative movement tracks, visually mapped for one replicate in each group, pre-exposure to SCW displayed linear movement, with most linear tracks spanning the field of view. Post-exposure to SCW, the tracks generated by the miracidia showed a clear change in linearity and displacement (Fig. 1). Post-exposure tracks had high levels of curvature across all treatment groups, representing miracidia changing direction. A reduced displacement of tracks was also observed due to miracidia spending more time in one location. No clear qualitative differences were observable in the tracks generated by miracidia exposed to SCW from different snail species. For the control groups, where miracidia were exposed to artificial pond water only, there were no visually observable behavioural changes over the duration of the experiment.

### Quantitative analysis of miracidial behaviour changes in response to snail-conditioned water

**Fig. 2:**
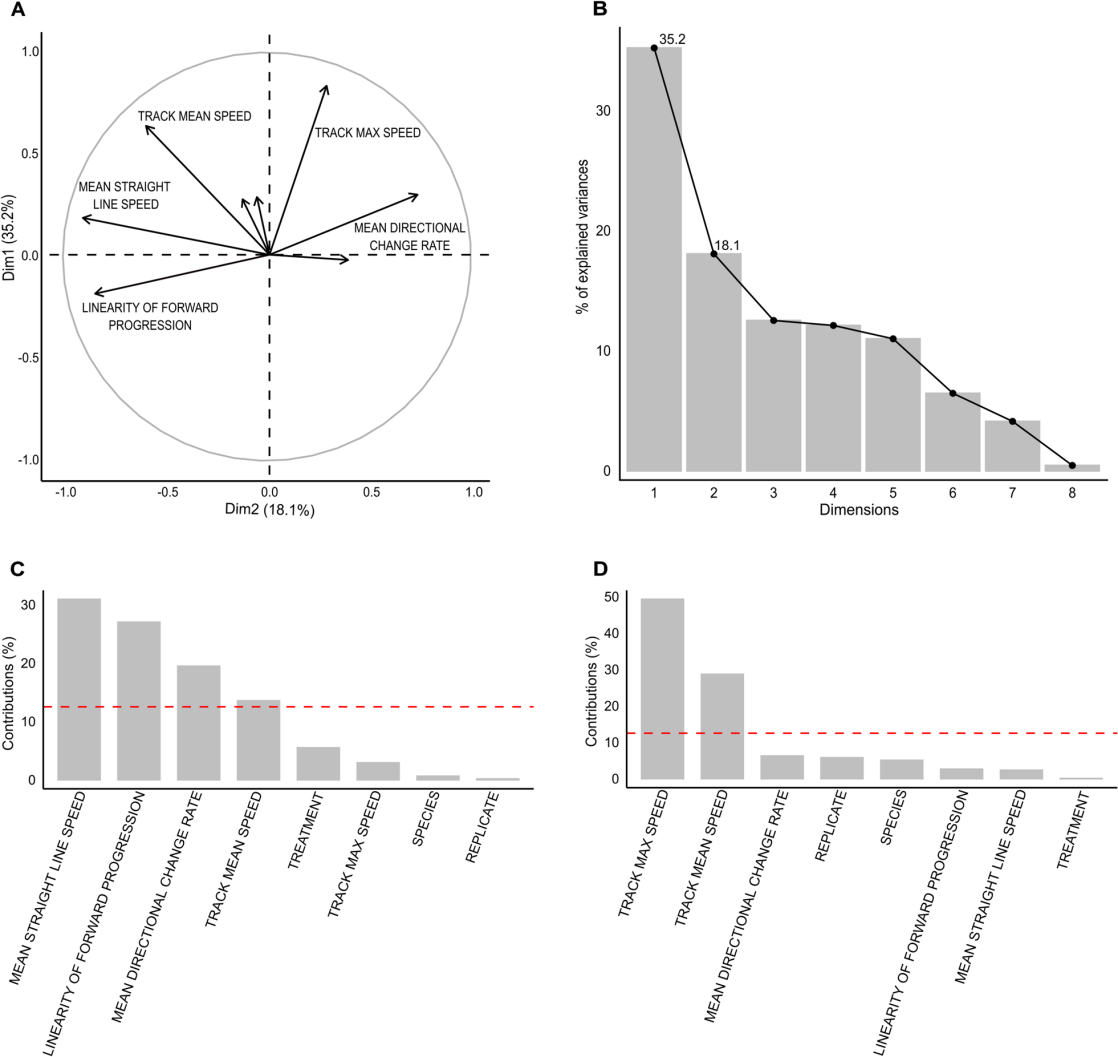
Principal component analysis plots generated in R, showing parameters contributing to variation in behavioural response to snail-conditioned water. PCA circle plot displays the correlation of each variable with dimensions 1 and 2, and the length of the arrow indicates its contribution level to each dimension. **A** Arrows pointing in the same direction are positively correlated. The scree plot describes the percentage contribution of each dimension to overall variability. **B** Histograms represent the contribution of variables within dimension 1 (**C**) and dimension 2 (**D**). The dashed red line indicates the average expected contribution; bars that cross the threshold contribute most highly to variation within the dimension

TrackMate analysis of video footage generated 25 track features for each miracidial track (Supplementary Table S1). Key features investigated were mean straight-line speed, linearity of forward progression, mean directional change rate, track mean speed and track maximum speed. To assess the importance of each track feature in explaining miracidial behavioural changes following exposure to SCW, a principal components analysis (PCA) was performed. The first and second dimensions explained 35.2% and 18.1% of the variation, respectively (Fig. 2A, B). Within dimension 1, the greatest contributions to the observed variation were mean straight-line speed (30.90%), linearity of forward progression (27.0%) and mean directional change rate (19.5%) (Fig. 2C). The greatest contributors to the observed variation in dimension 2 were track maximum speed (49.3%) and track mean speed (28.7%) (Fig. 2D). The greatest combined contributors to both dimensions were mean straight-line speed (21.2%) and linearity of forward progression (18.7%) (Supplementary Table S2). On the basis of these findings, mean straight-line speed (i.e. velocity) and linearity of forward progression (i.e. angularity) were chosen to report the changes in miracidial behaviour.

**Fig. 3:**
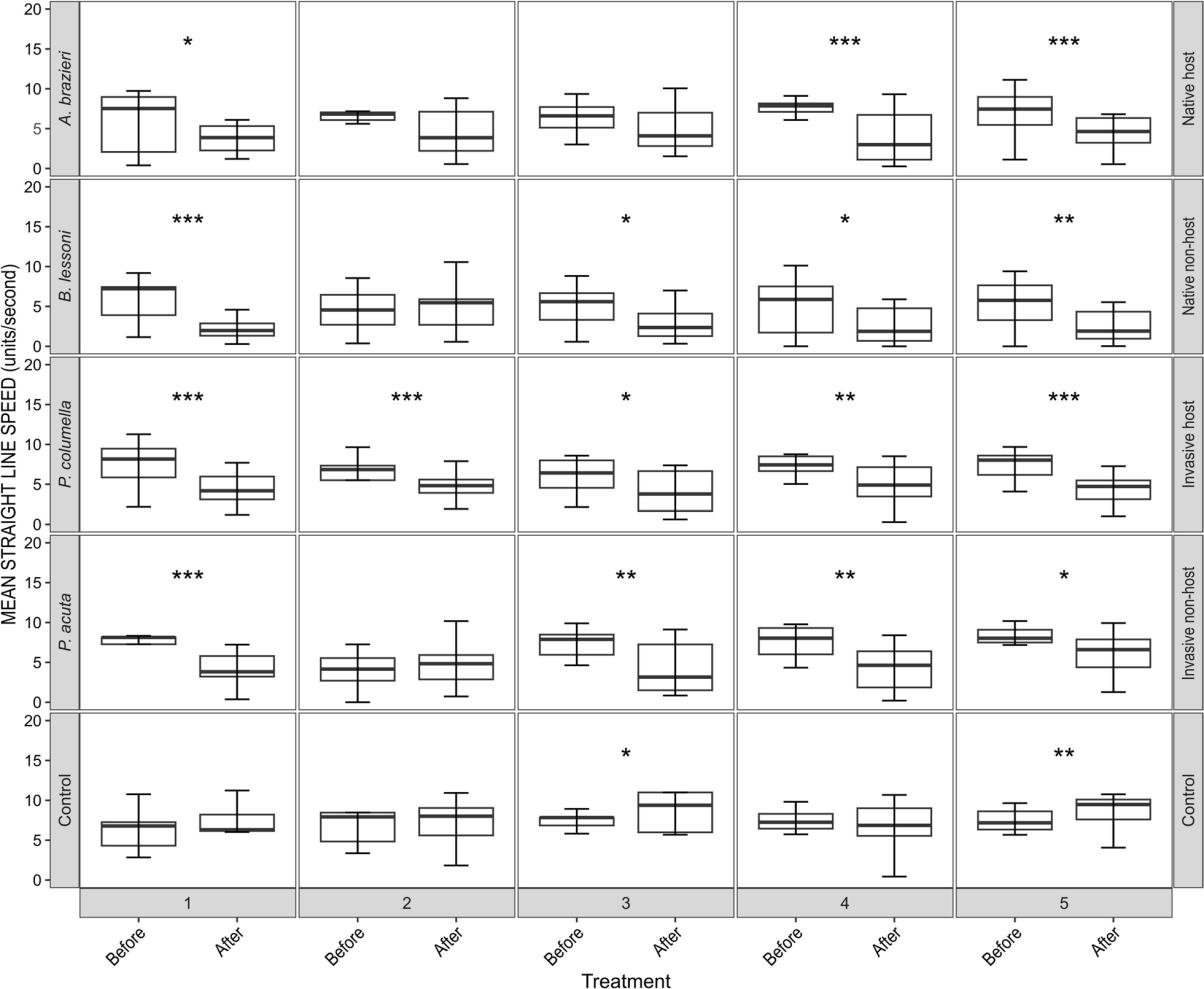
Change in mean straight-line speed of *Fasciola hepatica* miracidia within 1 min of exposure to native host (*Austropeplea* cf. *brazieri*) snail-conditioned water (SCW), native non-host (*Bullastra lessoni*) SCW, invasive host (*Pseudosuccinea columella*) SCW, invasive non-host (*Physa acuta*) SCW and artificial pond water. Each box plot pair represents one biological replicate. Boxes indicate the interquartile range (IQR), with the horizontal line denoting the median. Whiskers extend to the smallest and largest values within 1.5 × IQR, while data beyond this range are shown as outliers. *P*-values were calculated using the Mann–Whitney *U* test. ^*^P ≤ 0.05, ^**^P ≤ 0.01 and ^***^P ≤ 0.001

**Fig. 4:**
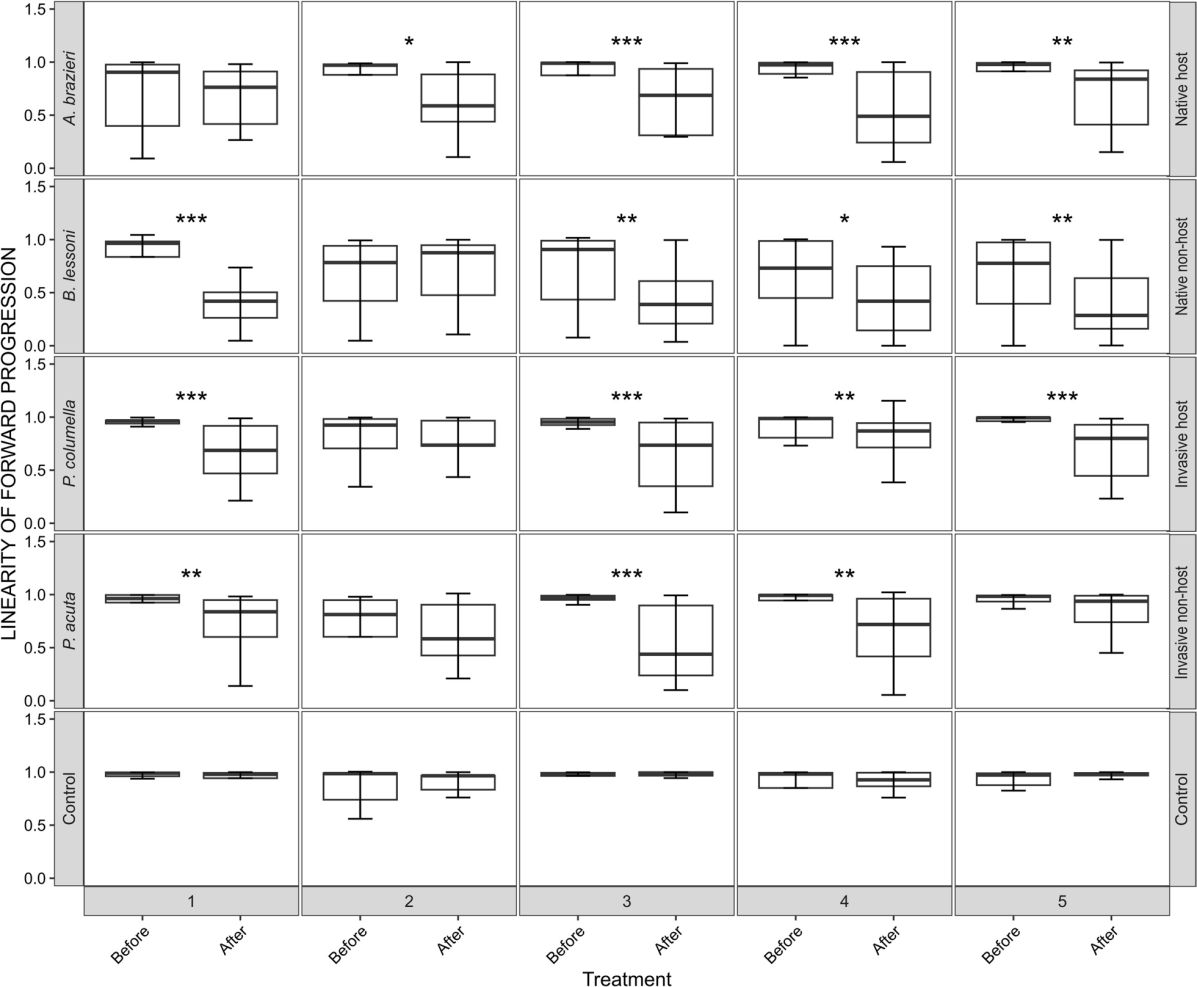
Change in linearity of forward progression of *Fasciola hepatica* miracidia within 1 min of exposure to native host (*Austropeplea* cf. *brazieri*) snail-conditioned water (SCW), native non-host (*Bullastra lessoni*) SCW, invasive host (*Pseudosuccinea columella*) SCW, invasive non-host (*Physa acuta*) SCW and artificial pond water. Each box plot pair represents one biological replicate. Boxes indicate the interquartile range (IQR), with the horizontal line denoting the median. Whiskers extend to the smallest and largest values within 1.5 × IQR, while data beyond this range are shown as outliers. *P*-values were calculated using the Mann–Whitney *U* test. ^*^P ≤ 0.05, ^**^P ≤ 0.01 and ^***^P ≤ 0.001

Both mean straight-line speed and linearity of forward progression were not normally distributed for all replicates (*P* <0.05, *W* values = 0.821–0.978; Shapiro–Wilk test; Supplementary Figure S1). Therefore, each miracidial behavioural track feature was compared before and after exposure to SCW using pairwise Mann–Whitney *U* tests (Figs. 3, 4). Mann–Whitney *U* tests indicated that there was a significant reduction in the mean straight-line speed of miracidia post-exposure to SCW for all snail species, although the effect was varied across replicates (Fig. 3). For *A.* cf. *brazieri* (native host), three of five replicates showed significant reductions (*P* ≤ 0.05, *W* = 310–370). For *B. lessoni* (native non-host), four of five replicates were significant (*P* ≤ 0.05, *W* = 306–403). For *P. columella* (invasive host), five of five replicates showed significant reductions (*P* ≤ 0.05, *W* = 309–418), and for *P. acuta* (invasive non-host), four of five replicates were significant (*P* ≤ 0.05, *W* = 314–374). Straight-line speed was measured in physical units determined by pixel size, and seconds as the units of the frame interval. The range of straight-line speed was 3.51–8.55 u/s across all replicates before SCW exposure. Post-exposure to SCW, the speed of the miracidia reduced to 1.66–6.60 u/s, with the greatest reduction in speed observed post-exposure to *A.* cf. *brazieri* (native host) SCW and the smallest reduction in speed observed in miracidia post-exposure to *P. acuta* (invasive non-host) SCW (Fig. 3). In contrast to this, there was a significant increase in the mean straight-line speed of two of five replicates in the control group post-exposure to artificial pond water (*P* ≤ 0.05, *W* = 108–134), with a pre-exposure range of 6.78–7.18 u/s increasing to 6.86–9.70 u/s post-exposure (Fig. 3).

The linearity of forward progression is a ratio, where a number closer to 1 indicates a more linear track. Mann–Whitney *U* tests also showed that the linearity of forward progression in the miracidia was significantly reduced in most SCW-exposed groups, representing greater angularity of tracks (Fig. 4). Significant reductions in linearity were observed in four out of five replicates for *A.* cf. *brazieri* (native host) (*P* ≤ 0.05, *W* = 322–362), *B. lessoni* (native non-host) (*P* ≤ 0.05, *W* = 304–386) and *P. columella* (invasive host) (*P* ≤ 0.01, *W* = 336–417). For *P. acuta* (invasive non-host), three of five replicates showed significant reductions in linearity (*P* ≤ 0.01, *W* = 332–382). Before exposure, the ratios for linearity of forward progression ranged from 0.73 to 1.00 across species, with an overall shift to a lower range of 0.29–0.95 post-exposure to SCW. The greatest changes were observed in the miracidia groups exposed to *B. lessoni* (native non-host) SCW, while the miracidia groups exposed to *P. columella* (invasive host) SCW showed the least change. There was only a minor, non-significant (*P* > 0.05) change in the control group, with a range of 0.90–0.99 pre-exposure and a range of 0.93–0.99 post-exposure to artificial pond water.

### Miracidia snail host attachment assay

The reduction in the number of miracidia in each well after the addition and removal of a snail was considered to be due to successful attachment to the snail’s soft tissue. Clear differences were observed in the numbers of miracidia absent from wells post-exposure to snails, with attachment rates ranging from 0.0% to 100.0% (Table 1). Wells with *A.* cf. *brazieri* (native host) and *B. lessoni* (native non-host) experienced the highest rate of attachment, with averages of 96.9% and 94.0%, respectively. The majority of miracidia exposed to *P. columella* (invasive non-host) successfully attached to the snail tissue, with an average attachment rate of 57.0%. For *P. acuta* (invasive non-host), successful attachment was only observed in one of the four snails exposed, resulting in a low average of 8.7% for the species (Table 1).

**Table 1:** Count of *Fasciola hepatica* miracidia before and after exposure to four freshwater snail species

	*Austropeplea* cf. *brazieri*	*Bullastra lessoni*	*Pseudosuccinea columella*	*Physa acuta*
	Native host	Native non-host	Invasive host	Invasive non-host
*N* (miracidia) at *T* = 0 min	26; 25; 22; 23^a,b^	24; 26; 20; 20	26; 21; 24; 22	20; 26; 22; 17
*N* (miracidia) at *T* = 30 min	0; 2; 0; 1	0; 1;0; 4	15; 7; 14; 5	20; 17; 22; 17
Average percentage (%) attached^c^ ± SD	96.9 ± 3.9	94.0 ± 9.5	57.0 ± 17.8	8.7 ± 17.3

SD, standard deviation;*T*, time

^a^Data points are presented for replicates 1–4 for each species in numerical order

^b^Counts per well were obtained through visual identification of miracidia in still frames obtained from video footage

^c^Reduction in the number of free-swimming miracidia, as a measure of successful attachment of the intermediate host tissue

## Discussion

Our investigation demonstrates the inability of *F. hepatica* miracidia to discriminate between host and non-host freshwater snails found in Australia during host-finding and host-attachment phases in the parasite life cycle. The results observed in this study identify an independent relationship between the host-finding response in *F. hepatica* miracidia and the host status of the corresponding aquatic snail in Australia. Non-specific behavioural responses and variability in attachment rates of miracidia highlight the disconnect between initial attraction, the downstream success of attachment and snail–parasite compatibility.

Miracidia are known to increase their rate of directional change and perform a “turnback” style of swimming when proximal to a snail host [28, 44–46]. The behavioural responses elicited in the results of this study are aligned with host-finding behaviours documented in *F. hepatica* and other digenean parasites [16, 47]. Before exposure to SCW, the miracidia display exploratory, searching behaviours characterised by fast, linear movement with minimal turning. Upon exposure, a distinct transition to a host-finding movement profile occurred, with significant reductions in velocity and linearity. A more “stop-and-turn” motion, indicative of an intensified search for a potential host in their vicinity, was visually represented in the tracking maps, where a highly angular movement profile was shown following exposure to SCW of all four snail species (Fig. 1). This suggests that non-specific semiochemical attractants, chemical signals that elicit behavioural responses in other organisms (e.g. glycoproteins, peptides and metabolites), act as recognition factors for these larvae [47–49].

Conditioned water from both host and non-host snails induced similar behavioural responses in *F. hepatica* miracidia, indicating that they are not able to infer the host status of the snail species from exposure to SCW alone. It is important to note that this study did not analyse the preference of miracidia between snail species when exposed to the SCW of more than one snail simultaneously. Exposure to a high concentration of compounds that trigger host-finding may cause miracidia to have an unnaturally strong response [28]. It is possible that, when exposed to a lower concentration or if given a choice between two gradients of SCW in a Y maze, miracidia would prefer one species over another.

Generalised attraction towards both host and non-host snail species would likely result in a lower infection rate in the environment, as miracidia expend time and energy to locate and attempt invasion of unsuitable hosts [50]. However, this feature may also have been a mechanism that facilitated the parasite’s success during invasion. *Fasciola hepatica* has coevolved with European lymnaeids, such as *Galba* truncatula, for thousands of years [51], and upon its introduction and exposure to novel host species in Australia, a nonspecific attraction to a range of snails would have been essential to try and complete its life cycle. The non-host-specific attraction to snail mucus that stimulates host-finding in *F. hepatica* may be advantageous for the parasite’s ability to survive in new ecological niches and adapt to the presence of novel species, situations likely to be relevant in the near future as the changing climate alters the distribution of currently occurring snail species [52, 53].

A combination of stimulatory molecules, physical configuration and chemical composition of the tissue surface is required for *F. hepatica* miracidia to attach and structurally alter the cells at the apical papilla for penetration into the musculature of the snail [43, 54, 55]. In miracidial studies of *Fascioloides magna*, non-host snail mucus was shown to have a larvicidal effect [56]. It is possible then that there are physiological differences between snail species which result in differences in miracidial attachment success; however, this has not been established in the snail species tested in this study. What has been evidenced is that, although miracidia had equally strong responses to the SCW of each snail species, the capacity for successful attachment to the tissue of the snail varied significantly between the four snail species tested and did not correlate with the suitability of the snail species as hosts for *F. hepatica*. Notably, almost 100% of miracidia were able to attach to native Australian lymnaeids, *A.* cf. *brazier* and *B. lessoni*, while only ~50% could attach to invasive *P. columella*. In contrast, miracidia exposed to invasive *P. acuta* showed a markedly lower attachment rate, with almost no individuals attaching successfully.

The absence of a relationship between attraction and attachment across all species suggests that species-specific factors inherent to the snail, more than parasite selectivity, determine the compatible status of the intermediate host. The observed differences between the two host species, *A.* cf. *brazieri* and *P. columella*, may be attributed to differences in host naïvety. Over time, coevolution of parasites and hosts results in the development of a suite of resistance mechanisms, as parasites attempt to maximise their infection potential and hosts attempt to ameliorate the associated morbidity [57–59]. The longer two organisms coexist with each other in the same environment, the higher the selection pressure for resistant phenotypes. This may explain the large difference in attachment rates between the two compatible host species. *Austropeplea* cf. *brazieri* is a relatively naïve host, having only been exposed to *F. hepatica* in the last ~200 years [10]. The American *P. columella* has been exposed to the parasite for at least twice this length of time, during the European colonisation of the Americas. In fact, some estimates based on ancient coprolites of Patagonian deer actually date the relationship to be more ancient than colonisation, at over 2000 years old [60]. The native *B. lessoni* is also a comparably naïve species and, similar to *A.* cf. *brazieri*, experienced attachment rates of nearly 100% but is an incompatible host for the parasite. It is possible, therefore, that an immune response from *B. lessoni*, absent in *A.* cf. *brazieri*, makes it an unsuitable host for *F. hepatica*. *Physa acuta*, which experienced the lowest rates of attachment, is a physid snail, a more distant species from the three lymnaeid snails. No physid snails are known hosts for *F. hepatica*, and they potentially possess physical differences, evolved over centuries, that impact the attachment success of miracidia [61, 62]. These findings highlight the complex and multifaceted interactions governing the relationship between the larval trematode and its host.

## Conclusions

The results of this study demonstrated that miracidia of *F. hepatica* were universally responsive to conditioned water from the snail species *A.* cf. *brazieri*, *B. lessoni*, *P. columella* and *P. acuta* under laboratory conditions. Additionally, miracidia attach to native Australian snails at a significantly higher rate than invasive snails, indiscriminate of host status. It appears that *F. hepatica* miracidia are capable of attraction and attachment to both host and non-host snail species under laboratory conditions, validating the hypothesis that *F. hepatica* miracidia lack pre-infection host-specificity at a species level in freshwater snail populations found in Australia. In future, exploring the specific mechanisms underpinning the invasion and development of *F. hepatica* within the snail host is warranted, using a combination of molecular and histopathological approaches.

## Supplementary Information

**Additional file 1: Figure S1.** Histograms of *Fasciola hepatica* miracidia behavioural response showing **(A)** change in mean straight-line speed and **(B)** change in linearity of forward progression before and after exposure to snail-conditioned water (SCW) or artificial pond water. Shapiro-Wilk test was used to determine the normality of the distribution. *P* < 0.05 indicates a non-normal distribution.

**Additional file 2: Table S1.** Raw sampled data of tracks generated from Trackmate.

**Additional file 3: Table S2.** Contributions of each variable to Dim-1 and Dim-2 of PCA.

## Data Availability

The authors confirm that the data supporting the findings of this study are available within the article [and/or] its supplementary materials.

## Abbreviation

SCW: Snail-conditioned water
