# Supplementary figures and images for: Intermediate host recognition abilities of Fasciola hepatica miracidia
Source: Parasit Vectors. 2025 Nov 25;18:489. doi: 10.1186/s13071-025-07125-y (PMC12648805; doi:10.1186/s13071-025-07125-y)

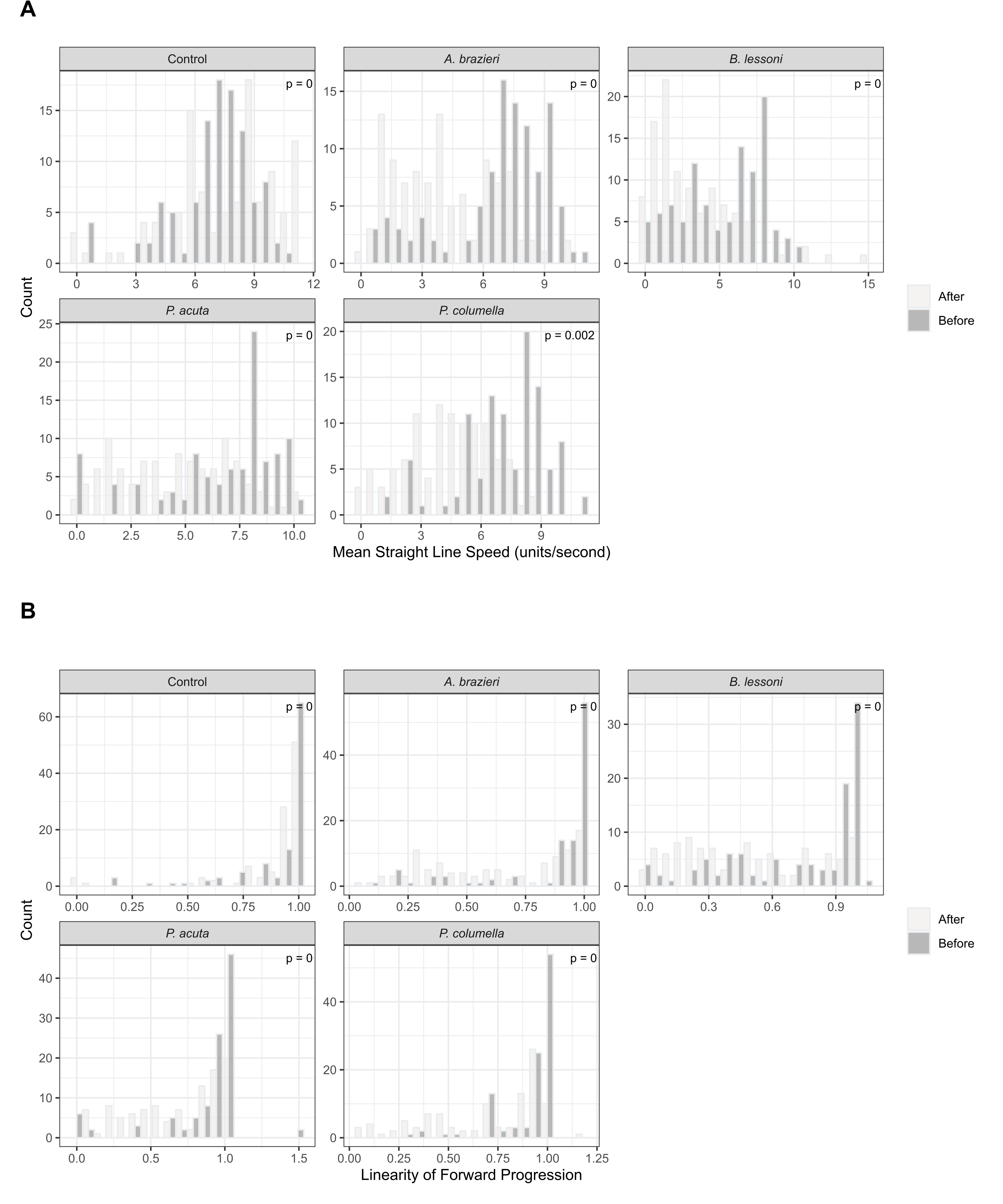

Supplement: Supplementary file 1 — Additional file 1: Figure S1. Histograms of Fasciola hepatica miracidia behavioural response showing (A) change in mean straight-line speed and (B) change in linearity of forward progression before and after exposure to snail-conditioned water (SCW) or artificial pond water. Shapiro-Wilk test was used to determine the normality of the distribution. P < 0.05 indicates a non-normal distribution. [file 13071_2025_7125_MOESM1_ESM.png]
